# Novel Signal-Amplified Fenitrothion Electrochemical Assay, Based on Glassy Carbon Electrode Modified with Dispersed Graphene Oxide

**DOI:** 10.1038/srep23409

**Published:** 2016-03-22

**Authors:** Limin Wang, Jinbo Dong, Yulong Wang, Qi Cheng, Mingming Yang, Jia Cai, Fengquan Liu

**Affiliations:** 1 College of Plant Protection (Key Laboratory of Integrated Management of Crop Diseases and Pests), Nanjing Agricultural University, Nanjing, 210095, P.R. China; 2 Institute of Plant Protection, Jiangsu Academy of Agricultural Science, Nanjing, 210014, P.R. China

## Abstract

A novel signal-amplified electrochemical assay for the determination of fenitrothion was developed, based on the redox behaviour of organophosphorus pesticides on a glassy carbon working electrode. The electrode was modified using graphene oxide dispersion. The electrochemical response of fenitrothion at the modified electrode was investigated using cyclic voltammetry, current-time curves, and square-wave voltammetry. Experimental parameters, namely the accumulation conditions, pH value, and volume of dispersed material, were optimised. Under the optimum conditions, a good linear relationship was obtained between the oxidation peak current and the fenitrothion concentration. The linear range was 1–400 ng·mL^−1^, with a detection limit of 0.1 ng·mL^−1^ (signal-to-nose ratio = 3). The high sensitivity of the sensor was demonstrated by determining fenitrothion in pakchoi samples.

Organophosphorus pesticides (OPs) are a major class of pesticides. They are widely used as pesticides, insecticides, and chemical-warfare agents^1^, and have high efficacy and toxicity. OPs have already become significant environmental and food-chain pollutants^2^. They inhibit acetylcholinesterase activity, leading to the accumulation of acetylcholine at nerve endings, resulting in damage to non-target organisms^3^. The illegal use of some OPs has seriously damaged the health of animals and humans^4^,^5^. The World Health Organization has classified OPs as “extremely hazardous” to the environment^6^. The use of OPs has raised serious public concern^7^,^8^, in terms of environmental safety, food safety, and human health, and OP detection is an important issue.

The most common methods for the determination of OPs are gas chromatography and high-performance liquid chromatography, sometimes coupled with mass spectrometry^9^,^10^,^11^,^12^,^13^. These methods are highly sensitivity, but they have disadvantages such as time-consuming sample preparation steps, expensive instrumentation, and the need for professional analysts. There is therefore a growing need for more rapid and economical methods for determining OPs.

Electrochemical detection techniques, which are low cost, can be used for field measurements, and are generally not time consuming^14^, have been widely studied. It has been reported that some OPs such as paraoxon, methyl parathion, and fenitrothion, which are nitroaromatic organophosphorus compounds, show good redox activities at electrode surfaces^15^,^16^. Many electrochemical studies of methyl parathion determination have been reported^17^,^18^,^19^. Wang *et al*.^20^ developed a glassy carbon electrode (GCE) coated with an acetylene black–chitosan composite film. They made an electrochemical sensor for detecting the irreversible reduction peak of methyl parathion, using a 30 s accumulation time under an open-circuit potential. The linear range was 5.26–26 320 ng·mL^−1^, with a detection limit of 0.5 ng·mL^−1^ (signal/noise ratio (*S*/*N*) = 3). Tan *et al*.^21^ electrodeposited a methyl parathion-templated molecularly imprinted porous silicate thin film on a GCE for methyl parathion detection. After accumulation for 80 s, the oxidation peak of methyl parathion was scanned with the sensor; the linear range was 2.63–2632 ng·mL^−1^, and the detection limit was 2.34 ng·mL^−1^ (*S*/*N* = 3). Yang *et al*. used Au–ZrO_2_–SiO_2_ nanocomposite spheres to modify an electrode for OP (paraoxon-ethyl) extraction^22^. The electrode was dipped for in the sample solution for 5 min, and the oxidation peak current was recorded. The response was linear in the concentration range 1–500 ng·mL^−1^, and the detection limit was 0.5 ng·mL^−1^. Liu used a carbon paste electrochemical transducer to analyse organophosphates^1^. A linear relationship between the stripping current and methyl parathion concentration was obtained in the concentration range 263.21–15 792.6 ng·mL^−1^, after adsorption for 2 min, and the detection limit was 13.16 ng·mL^−1^ after adsorption for 10 min.

In this paper, we describe a novel electrochemical method for detecting OPs (fenitrothion was used as a model); this method greatly enhances the electrical signal from fenitrothion (Fig. 1). The graphene oxide dispersion was used to modify the electrode. The modification procedure was very simple and convenient; it involved pipetting a given quantity of graphene oxide dispersion onto the surface of a clean, dry GCE. The detection steps were as follows. Square-wave voltammetry was used to scan the irreversible reduction peak from −0.2 to −1.0 V after a given accumulation time. After accumulation for another optimum time, square-wave voltammetry scanning was performed from −0.6 to 0.3 V to record the oxidation peak, for detection and quantification of fenitrothion. A good linear relationship between the oxidation peak current and the fenitrothion concentration was obtained under the optimised conditions. The linear range was 1–400 ng·mL^−1^, with a detection limit of 0.1 ng·mL^−1^ (*S*/*N* = 3). The high sensitivity of the method was demonstrated by determining fenitrothion in pakchoi samples. The results show that this is a reliable method for the determination of OPs, and has good potential for practical applications.

## Results and Discussion

### Electrode characterisation

Figure 2A,B show the SEM images of top views of two different electrodes. The bare GCE (Fig. 2A) has a smooth surface. The surface of the GCE with dispersed graphene oxide is rough (Fig. 2B). These results confirm that the electrode surface was successfully modified by simply pipetting the graphene oxide dispersion onto the GCE.

The successful of the grapheme oxide dispersion onto the GCE was further confirmed by EDS spectra as shown in Fig. 2C,D. It shows that the only C presence on bare GCE and C, O presence on graphene oxide modified GCE, which confirmed that the loading of graphene oxide on GCE was successful.

### Electrochemical response of fenitrothion on GCE modified with graphene oxide

Figure 3A shows cyclic voltammograms of 5 μg·mL^−1^ fenitrothion in an acetate buffer at the bare GCE (curve b) and at the GCE modified with dispersed graphene oxide (curve c). A weak signal is observed at the bare GCE in the potential range −0.9 to 0.4 V; the irreversible reduction peak at −0.55 V and two peaks at about 0.024 V are clearly stronger with the modified GCE. These peaks are assigned to be the signal from the redox reaction of fenitrothion, because analogous signals (curve a) are not observed for either electrode in the absence of fenitrothion under the same conditions.

**Figure 1:**
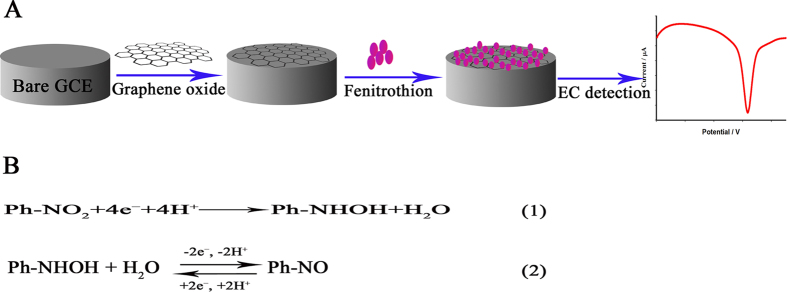
Schematic of electrochemical detection of fenitrothion based on graphene oxide modified GCE (**A**) and the equation for the mechanism of the electrochemical reaction (**B**).

**Figure 2:**
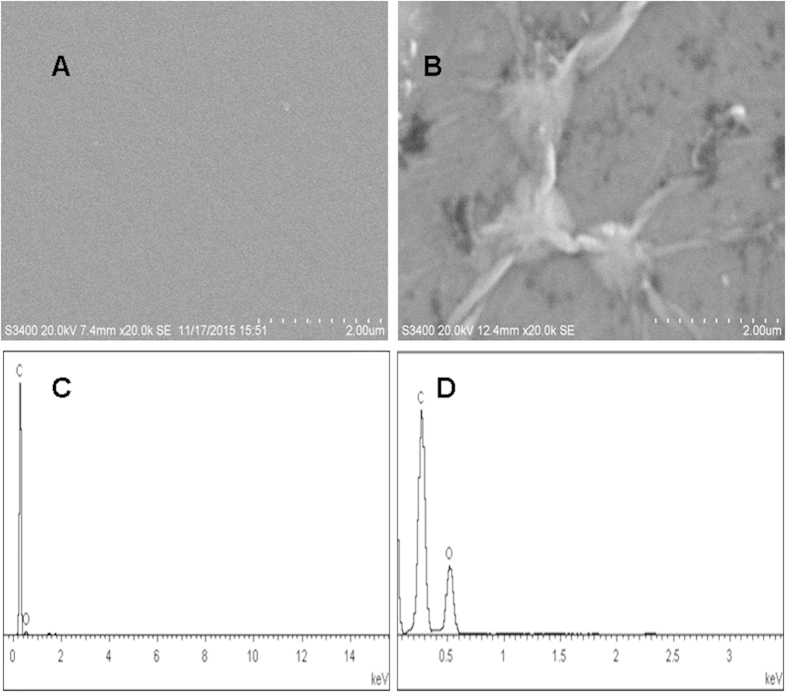
SEM images of bare GCE (**A**) and GCE modified with graphene oxide dispersion (**B**), the EDS of bare GCE (**C**) and GCE modified with grapheme oxide dispersion (**D**).

**Figure 3:**
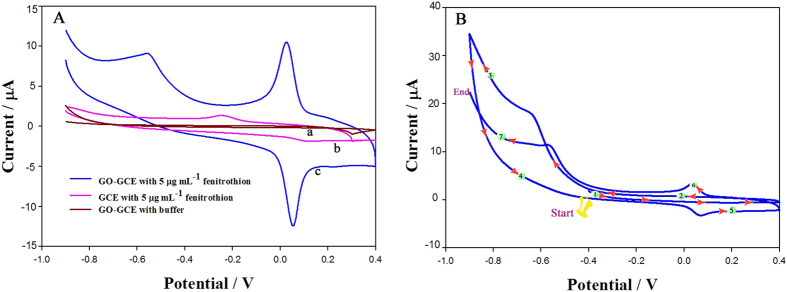
(**A**) Cyclic voltammograms of buffer (a), 5 μg·mL^−1^ fenitrothion at bare GCE (b), and 5 μg·mL^−1^ fenitrothion at GCE modified with graphene oxide dispersion (c); (**B**) Electrochemical response of 5 μg·mL^−1^ fenitrothion at modified GCE, based on cyclic voltammetry.

The mechanism of this electrochemical reaction was studied using cyclic voltammetry. Figure 3B shows the electrochemical response without accumulation. There is no oxidation peak (P_a_) and reduction peak (P_c1_) in the first cycle. The electrochemical response of fenitrothion is the same as that of methyl parathion; it starts with an irreversible reduction peak (P_c_) generated by the nitrophenyl group capturing four electrons to form hydroxylamine (equation 1 in Fig. 1). In the second cycle, the reversible peaks (P_a_ and P_c1_) are formed via two-electron transfer between hydroxylamine and a nitroso group (equation 2 in Fig. 1). These reactions are consistent with other results reported for OPs and nitroaromatic compounds^20^,^21^,^22^,^23^,^24^,^25^,^26^. A two-step method was therefore used: the irreversible reduction peak was scanned from −0.2 to −1.0 V, and then the oxidation peak, which appeared at 0.024 V, was recorded, for detection and quantification of fenitrothion. The oxidation peak current increased with increasing scan rate and was linearly correlated with the scan rate from 10 to 400 mV·s^−1^ (Figure S1).

### Optimisation of experimental conditions

#### Effect of electrolyte

Citric acid-sodium citrate buffer, glycine–HCl buffer, phosphate-buffered solution, and acetate buffer were used as supporting electrolyte solutions for fenitrothion detection. Acetate buffer was selected as the supporting electrolyte (Figure S2), based on the peak shape, signal strength, and electrode stability.

#### Effect of accumulation conditions

**Figure 4:**
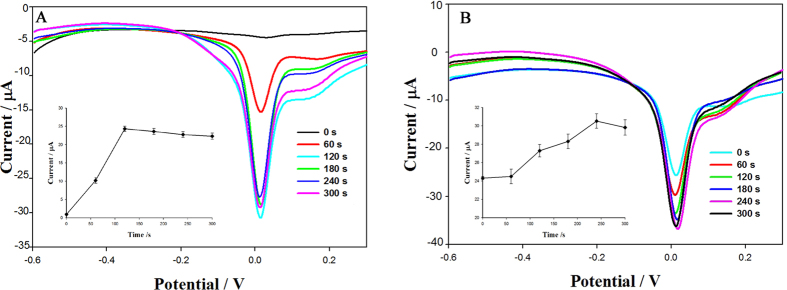
Effect of accumulation time of (**A**) irreversible reduction and (**B**) oxidation-reduction on peak current of 250 ng·mL^−1^ fenitrothion at GCE modified with graphene oxide dispersion.

The effect of the accumulation time on the peak current for 250 ng·mL^−1^ fenitrothion at the GCE modified with a graphene oxide dispersion was investigated (Fig. 4). Accumulation times of 0 to 300 s were used. After accumulation, the irreversible reduction peak was scanned, and then the oxidation peak was scanned without accumulation, according to the maximum oxidation peak current. An accumulation time for irreversible reduction of 120 s was chosen (Fig. 4A), and was used to investigate the accumulation time for the oxidation peak. As shown in Fig. 4B, the maximum oxidation peak was obtained after accumulated for another 240 s. Without any accumulation times (0 s for both the irreversible reduction peak and the oxidation peak), the current signal was very weak. Accumulation times of 120 s for the irreversible reduction peak and 240 s for the oxidation peak gave the maximum oxidation peak current. Scanning therefore involved accumulation for 120 s under an open-circuit potential, recording the irreversible reduction peak, accumulation for another 240 s under the same potential, and recording the oxidation peak.

#### Effect of pH

**Figure 5:**
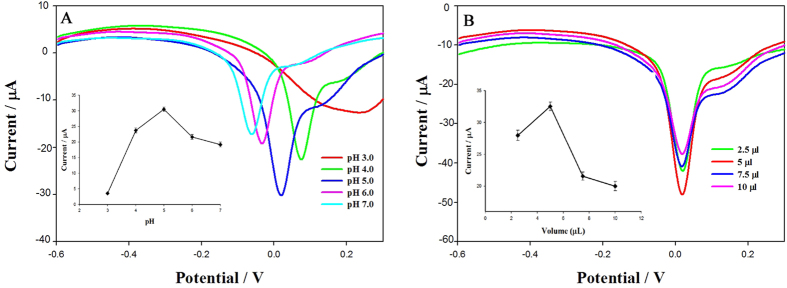
(**A**) Effect of pH on peak current of 250 ng·mL^−1^ fenitrothion at GCE modified with graphene oxide dispersion; (**B**) Effect of graphene film thickness on peak current of 250 ng·mL^−1^ fenitrothion at GCE modified with graphene oxide dispersion.

The effect of pH on the oxidation signal in acetate buffer was studied in the pH range 3.0–7.0; the results are shown in Fig. 5A. The current signal increased with increasing solution pH value to pH 5.0, and then decreased between pH 5.0 and 7.0. These results indicate that weak acidic conditions are suitable for the oxidation reaction. In contrast, a strong acid buffer resulted in weak signal strength and poor peak shape; pH 5.0 was selected as the optimum pH.

#### Effect of graphene film thickness

The graphene film thickness also influenced fenitrothion determination. As shown in Fig. 5B, the sensitivity of the sensor increased with increasing volume of graphene dispersion, until reaching a maximum at 5 μL. When the volume exceeded 5 μL, the current signal decreased. Graphene oxide is a porous-structured material, the graphene oxide film on GCE would increase the signal and sensitivity. However, the film thickness is increasing with the graphene oxide further adding. The graphene oxide film would be too thick to block of electron transfer on the GCE.

### Comparison of two detection methods using different oxidation peak scanning methods

**Figure 6:**
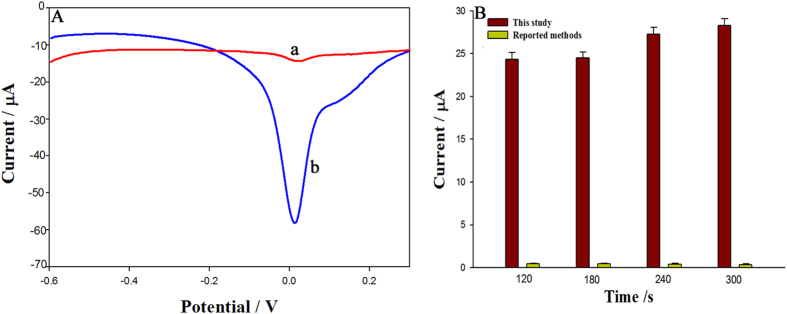
(**A**) Oxidation peak signal of 400 ng·mL^−1^ fenitrothion scanned using (a) detection way reported and (b) detection way this study developed at GCE modified with graphene oxide dispersion; (**B**) Oxidation peak currents for two detection methods with same accumulation times (total).

The signal strengths for 400 ng·mL^−1^ fenitrothion obtained using a previously reported^21^,^22^,^24^ detection method and our method, under the respective optimised conditions, were compared. As shown in Fig. 6A, for our method, the scanned oxidation peak signal after the irreversible reduction peak is stronger than that obtained by scanning the oxidation peak directly. This indicates that our method improves signal detection. We also wanted to determine whether the different accumulation times used in the two methods led to different signal strengths. Figure 6B shows the oxidation peak currents achieved by the two methods, using the same accumulation times. For the previously reported method, the accumulation time was calculated by combining the irreversible reduction peak accumulation time and oxidation peak accumulation time. It is clear that the signals differ significantly, even with the same accumulation times. This proves that our method enhances the signal strength because of the detection steps used, not because of the long accumulation time.

Futhermore, the signal strengths for 400 ng·mL^−1^ parathion and paraoxon were also obtained using the previously reported detection method and our method. As shown in Figure S3, for our method, the scanned oxidation peak signal for parathion and paraoxon after the irreversible reduction peak is also stronger than that obtained by scanning the oxidation peak directly.

### Analytical application

**Figure 7:**
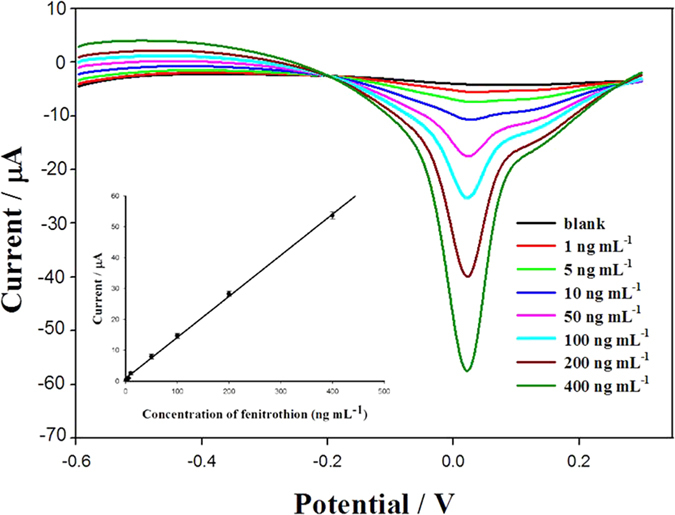
Square-wave voltammograms of acetate buffer (**a**), and 1 ng·mL^−1^ (**b**), 5 ng·mL^−1^ (**c**), 10 ng·mL^−1^ (**d**), 50 ng·mL^−1^ (**e**), 100 ng·mL^−1^ (**f**), 200 ng·mL^−1^ (**g**), and 400 ng·mL^−1^ (**h**) fenitrothion at GCE modified with graphene oxide dispersion. The inset is the calibration curve for fenitrothion determination.

A series of fenitrothion solutions of different concentration were analysed using our detection method under the optimum conditions. A good linear relationship between the peak current and concentration was obtained, as shown in Fig. 7; the linear range was 1–400 ng·mL^−1^. The linear equation is expressed as *i*_pa_ (μA) = 0.133*c* (ng mL^−1^) + 0.953 (*R*^2^ = 0.999). The detection limit was 0.1 ng·mL^−1^, based on *S*/*N* = 3.

The reproducibility was evaluated based on intra- and inter-assay coefficients of variation (CVs). The intra-assay was performed by analysing 400 ng·mL^−1^ fenitrothion six times on the same graphene oxide-modified GCE. The intra-assay CV was 4.3%. The inter-assay was performed by analysing 400 ng·mL^−1^ fenitrothion independently on six different electrodes; the CV was 7.8%.

The stability of the modified electrode was also investigated. The current response of the fenitrothion oxidation reaction started to decrease after eight electrochemical detection times. The modified electrode was stable at room temperature in the absence of air. Oxygen-containing inorganic ions such as NO_3_^−^, CO_3_^2−^, PO_4_^3−^, and SO_4_^2^ did not interfere with fenitrothion detection. These results indicate that the GCE modified with graphene oxide dispersion gives acceptable reproducibility, stability, and reliability.

### Determination of fenitrothion in pakchoi sample

To evaluate the practical performance of the developed two-step detection method, fenitrothion was added to pakchoi samples. Samples were prepared as described in part of Preparation of pakchoi samples of Materials and methods. The graphene oxide-modified GCE was immersed in the sample diluent (4 mL) for fenitrothion detection. Each concentration was examined three times. The results, which are summarised in Table 1, show that our two-step method gave good reliability in pakchoi sample analysis.

## Conclusion

A novel sensitive accumulation method for the electrochemical determination of fenitrothion was successfully developed. The electrochemical behaviour of fenitrothion on a GCE modified with dispersed graphene oxide was investigated thoroughly. Under the optimum conditions, we obtained a good linear relationship between the oxidation peak current and the fenitrothion concentration. The linear range was 1–400 ng·mL^−1^, with a detection limit of 0.1 ng·mL^−1^ (*S*/*N* = 3). A comparison of a one-step detection method with our two-step method showed that our method gave a much stronger signal. These results indicate that our method has high sensitivity for fenitrothion determination in solution (Table 2), and good reliability for fenitrothion detection in pakchoi samples. Our two-step method using a GCE modified with the graphene oxide dispersion is therefore promising for fenitrothion detection in environmental pollutants.

## Materials and Methods

### Materials and apparatus

Fenitrothion was purchased from the Shanghai Pesticide Research Institute (Shanghai, China). A 5 mg·mL^−1^ stock solution in methanol was prepared and stored at 4 °C. An aqueous solution was prepared by simple dilution of the stock solution with acetate buffer (pH 5.0). The graphene oxide dispersion (Figure S4) was bought from the Nanjing XFNANO Materials Tech Co., Ltd. (Nanjing, China). All other chemicals were of analytical grade, and all solutions were prepared using ultrapure water.

Electrochemical measurements were performed using a CHI660D electrochemical workstation (Chen Hua Instrument Co., Ltd., Shanghai, China) with a conventional three-electrode system consisting of a GCE working electrode, a Pt wire auxiliary electrode, and a saturated calomel reference electrode. The surface of the GCE modified with a graphene oxide dispersion was examined using scanning electron microscopy (SEM). SEM was performed by the Beijing ZKBC Testing and Technology Co., Ltd.

### Electrode modification with graphene oxide dispersion

A GCE was carefully polished with 0.3, 0.1, and 0.05 μm alumina slurries in succession, to give a mirror-like surface, and sequentially thoroughly sonicated in nitric acid (v/v = 1:1), ethanol, and redistilled water. The dried electrode was tested in 0.1 M KCl solution containing 0.5 mmol·L^−1^ K_4_[Fe(CN)_6_]/K_3_[Fe(CN)_6_] to ensure that it was sufficiently smooth and could be used for the experiments. Graphene oxide dispersion (5 μL, 0.5 mg·mL^−1^) was pipetted onto the surface of the clean, dry GCE. The electrode was dried at room temperature to obtain a GCE modified with dispersed graphene oxide as the working electrode.

### Electrochemical measurements

Fenitrothion detection was performed in a semi-hermetic electrochemical cell, with acetate buffer (pH 5.0, 4 mL). Detection involved the following two steps. (1) After accumulation for 120 s under an open-circuit potential, a square-wave voltammogram was recorded with potential sweeping from −0.2 to −1.0 V, a step potential of 4 mV, amplitude of 25 mV, and frequency of 25 Hz. (2) After accumulated for another 240 s under the same potential, a square wave voltammogram was recorded with potential sweeping from −0.6 to 0.3 V, a step potential of 4 mV, amplitude of 25 mV, and frequency of 25 Hz. The oxidation peak was used to quantify the fenitrothion in the supporting electrolyte solution. The electrochemical experiments were performed at room temperature.

### Method for oxidation peak scanning to quantify fenitrothion

It has previously been reported that OPs can be quantified by scanning the oxidation peak immediately after a given accumulation/adsorption time^21^,^22^,^24^. Unlike the case in our method, only one accumulation time was used. The materials and procedures used to modify the electrode in these other studies were different from those we used. We compared the signal strengths obtained using our electrochemical detection method with those obtained using a previously reported method. The optimum conditions, linear range, and detection limit were determined; the results are given in the Supporting Information (Figure S5 and S6).

### Preparation of pakchoi samples

Pakchoi samples were bought from a local market. First, the matrix effect was investigated. The pakchoi was cut into small pieces and placed in a 50 mL centrifuge tube; a certain amount of methanol was added. After thorough mixing, the sample was left to stand at room temperature overnight. Water (5 mL) and acetonitrile (30 mL) were added; the mixture was vortexed for 5 min, and sonicated for 15 min. Anhydrous magnesium sulfate (2 g) and NaCl (3 g) were added to the sample with stirring, and the mixture was vortexed for 1 min. The sample was then centrifuged at 4000 *g* for 15 min to separate the organic and water phases. The upper, organic phase (15 mL) was filtered through a funnel with anhydrous Na_2_SO_4_ blocked by absorbent cotton; the filtrate was steam rotary evaporated until no liquid was left. Then working buffer (5 mL) was added, using a pipette, to dilute the matrix 2-fold. Matrix concentration dilutions of 10×, 20×, and 40× resulted in low matrix effects. The initial amount of fenitrothion spiked into the sample was selected to give an 80× diluted matrix with a theoretically calculated value in the linear range. Fenitrothion determination in pakchoi samples was performed using the steps described above, apart from changing the amount of methanol so that it equalled the amount of standard fenitrothion solution, with final 80× dilution with working buffer.

## Additional Information

**How to cite this article**: Wang, L. *et al*. Novel Signal-Amplified Fenitrothion Electrochemical Assay, Based on Glassy Carbon Electrode Modified with Dispersed Graphene Oxide. *Sci. Rep.* **6**, 23409; doi: 10.1038/srep23409 (2016).

## Supplementary Material

Supplementary Information

## Figures and Tables

**Table 1:** Results for determination of fenitrothion in pakchoi samples.

Sample	Spiked (μg·g^−1^)	Found (μg·g^−1^)			Averge ± SD (μg·g^−1^)	Recovery ± RSD (%)
Pakchoi	5.0	5.04	4.85	4.67	4.85 ± 0.19	97.00 ± 3.81
	9.0	7.31	7.75	7.49	7.52 ± 0.22	83.56 ± 2.94
	12.0	12.15	11.4	11.91	11.82 ± 0.38	98.50 ± 3.24

**Table 2:** The performance of electrochemical detection methods was compared for the determination of organophosphorus pesticides.

Scanning method	Scanning peak	Modification method	Accumulation/adsorption time	Linear range (ng·mL^−1^)	Detection limit (ng·mL^−1^)	Comments	Reference
DPV	Pc	Acetylene black-chitosan composite film	30 s	5.26–26 320	0.5	Short accumulation time, more active sites, high sensitivity.	20
SWV	Pa	Molecularly imprinted sol-gel film	80 s	2.63–2632	2.34	Fast response, low detection limit, wide linear range, high selectivity and sensitivity	21
SWV	Pa	Au-ZrO_2_-SiO_2_ nanocomposite spheres	5 min	1–500	0.5	Fast, sensitive, and selective.	22
SWV	Pa	Gold/sodium dodecyl-benzene- sulfonate nanoparticles	120 s	131.61–26 321	22.64	Simple and convenient compared with self-assembled monolayers; easier and more controllable than common electrode modification techniques.	24
SWV	Pc and Pa	Graphene oxide dispersion	120 s + 240 s	1–400	0.1	Modification procedure is simple and convenient, electrical signal is enhanced, high sensitivity potential application prospect.	This study
