# Supplementary material for: Novel Signal-Amplified Fenitrothion Electrochemical Assay, Based on Glassy Carbon Electrode Modified with Dispersed Graphene Oxide
Source: Sci Rep. 2016 Mar 22;6:23409. doi: 10.1038/srep23409 (PMC4802211; doi:10.1038/srep23409)
Supplement: Supplementary Information [file srep23409-s1.doc]

**Supporting Information**

Novel Signal-Amplified Fenitrothion Electrochemical Assay, Based on Glassy Carbon Electrode Modified with Dispersed Graphene Oxide

Limin Wang^1,+,*^, Yulong Wang^1,+^, Jinbo Dong^1,+^, Qi Cheng^1^, Mingming Yang^1^, Jia Cai^1^, Fengquan Liu^1,2,*^

^1^ College of Plant Protection (Key Laboratory of Integrated Management of Crop Diseases and Pests), Nanjing Agricultural University, Nanjing, 210095, P.R.China

^2^ Institute of Plant Protection, Jiangsu Academy of Agricultural Science, Nanjing, 210014, P.R.China

* Correspondence and request for materials should be addressed to L.W. ([wlm@njau.edu.cn](mailto:wlm@njau.edu.cn)) or F.L. ([fqliu20011@sina.com](mailto:fqliu20011@sina.com))

^+^ The first three authors contributed equally to this work.


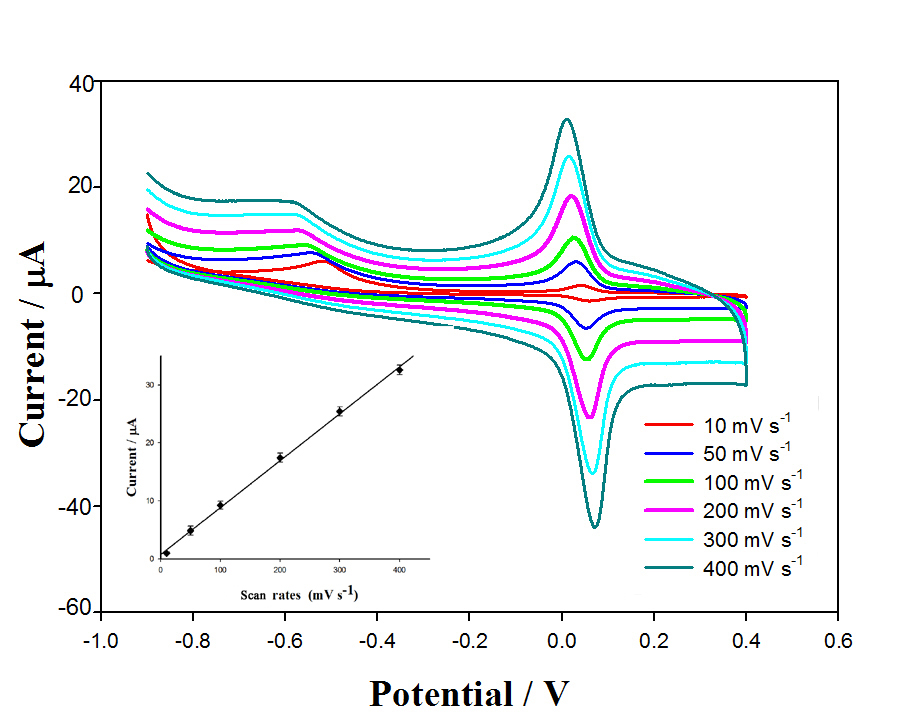


Figure S1. Cyclic voltammograms of 5 μg·mL^−1^ fenitrothion at GCE modified with graphene oxide dispersion at different sweep rates (from inner to outer: 10, 50, 100, 200, 300, and 400 mV s^−1^). Inset: plot of relationship between oxidation peak current and scan rate.


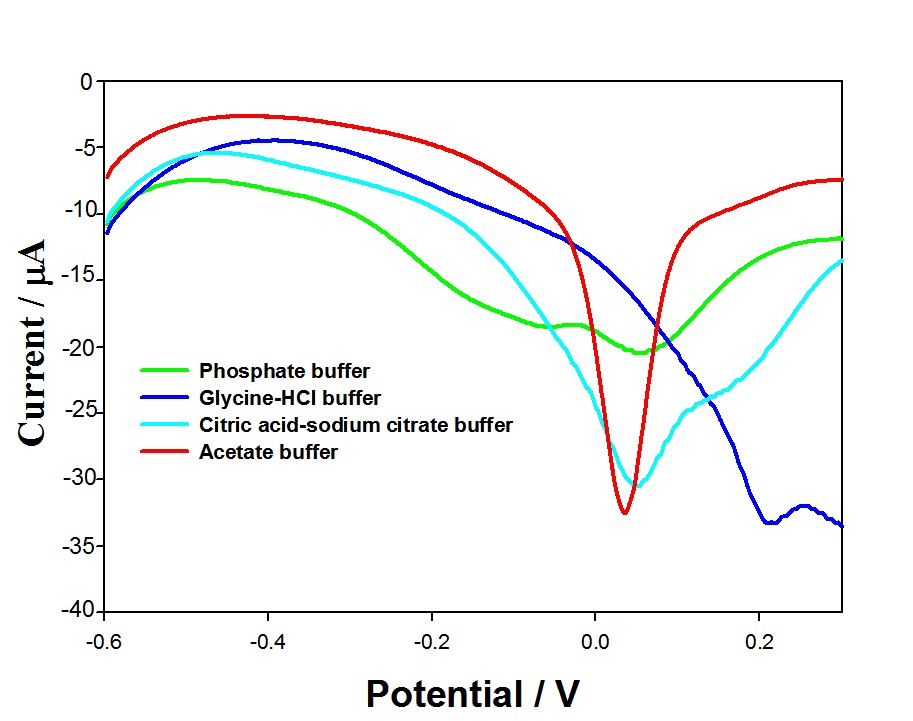


Figure S2 Effect of electrolyte on peak current of 250 ng·mL^−1^ fenitrothion at GCE modified with graphene oxide dispersion.

Figure S3: Oxidation peak signal of 400 ng·mL^−1^ Parathion (A) and Paraoxon (B) scanned using reported detection method and this study developed detection method at GCE modified with graphene oxide dispersion;

Figure S4: The XRD of graphene oxide dispersion. In the XRD pattern of GO, a strong peak was observed at 2θ=10.5°


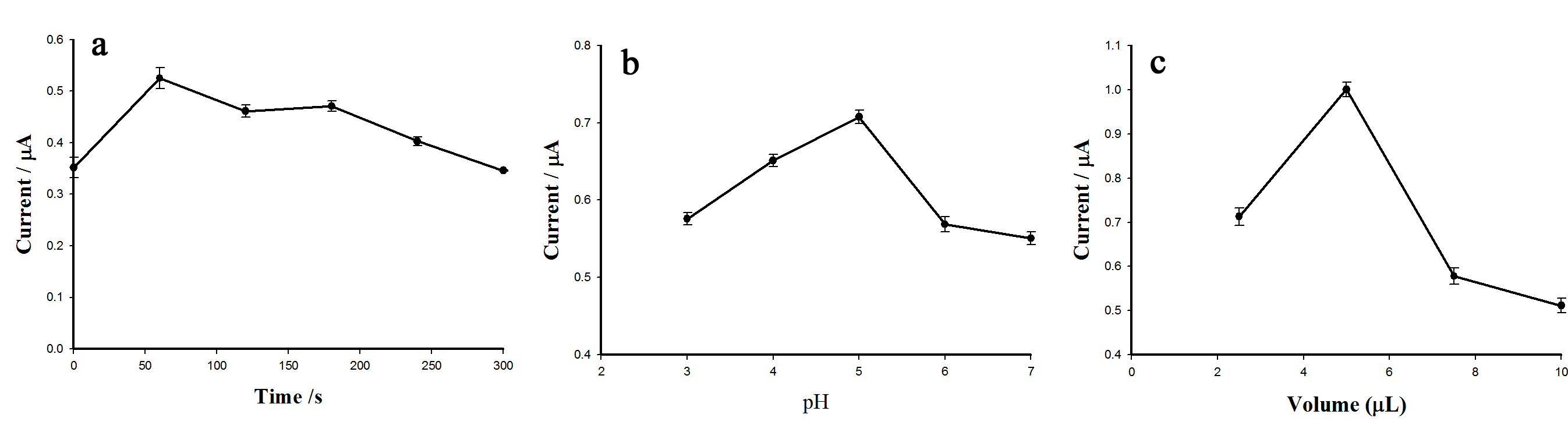


Figure S5. Influence of (a) accumulation time, (b) pH, and (c) graphene film thickness on peak current of 400 ng·mL^−1^ fenitrothion at GCE modified with graphene oxide dispersion, using reported method for scanning oxidation peak.

**
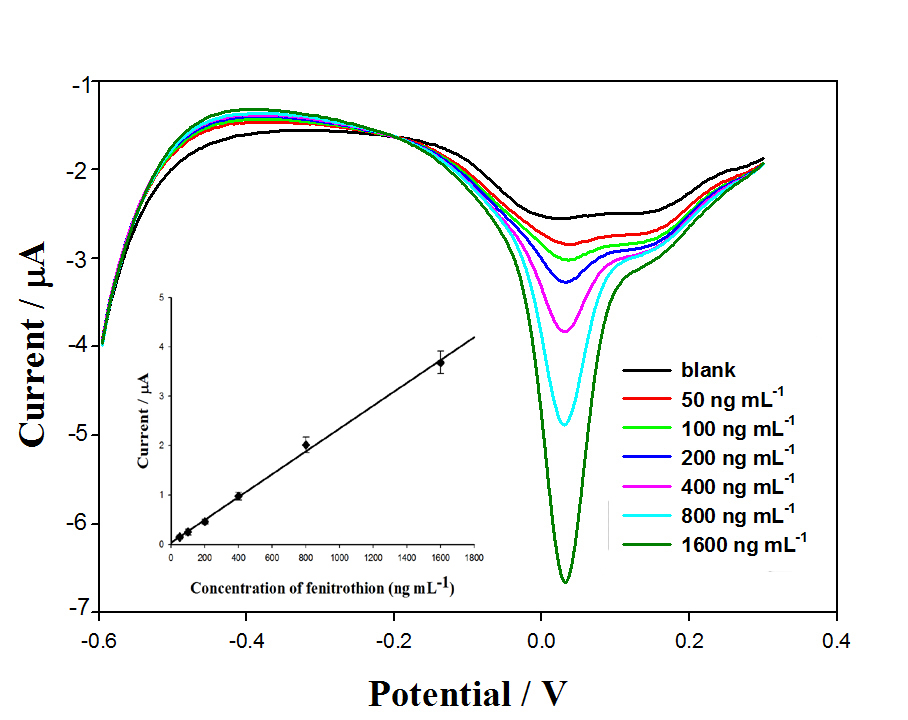
**

Figure S6. Scanning square-wave voltammograms, obtained using reported detection method, of acetate buffer (a), and 50 ng·mL^−1^ (b), 100 ng·mL^−1^ (c), 200 ng·mL^−1^ (d), 400 ng·mL^−1^ (e), 800 ng·mL^−1^ (f), and 1600 ng·mL^−1^ (g) fenitrothion at GCE modified with graphene oxide dispersion. Inset is calibration curve for fenitrothion determination.
